# A cross-sectional study of the filarial and *Leishmania* co-endemicity in two ecologically distinct settings in Mali

**DOI:** 10.1186/s13071-017-2531-8

**Published:** 2018-01-08

**Authors:** Moussa Brema Sangare, Yaya Ibrahim Coulibaly, Siaka Yamoussa Coulibaly, Michel Emmanuel Coulibaly, Bourama Traore, Ilo Dicko, Ibrahim Moussa Sissoko, Sibiry Samake, Sekou Fantamady Traore, Thomas Bruce Nutman, Jesus Gilberto Valenzuela, Ousmane Faye, Shaden Kamhawi, Fabiano Oliveira, Roshanak Tolouei Semnani, Seydou Doumbia

**Affiliations:** 1 International Center of Excellence in Research, Faculty of Medicine and Odontostomatology, Point G, Bamako, Mali; 2 National Institutes of Health, Bethesda, MD 20892 USA; 3 National Institutes of Health, Rockville, MD 20852 USA; 4 Centre National d’Appui à la lutte contre la Maladie (CNAM), Bamako, Mali

**Keywords:** Filariae, *Mansonella perstans*, *Wuchereria bancrofti*, *Leishmania major*, Co-endemicity, Vector-borne diseases, Mali

## Abstract

**Background:**

Filariasis and leishmaniasis are two neglected tropical diseases in Mali. Due to distribution and associated clinical features, both diseases are of concern to public health. The goal of this study was to determine the prevalence of co-infection with filarial (*Wuchereria bancrofti* and *Mansonella perstans*) and *Leishmania major* parasites in two ecologically distinct areas of Mali, the Kolokani district (villages of Tieneguebougou and Bougoudiana) in North Sudan Savanna area, and the district of Kolondieba (village of Boundioba) in the South Sudan Savanna area.

**Methods:**

The prevalence of co-infection (filarial and *Leishmania*) was measured based on (i) *Mansonella perstans* microfilaremia count and/or filariasis immunochromatographic test (ICT) for *Wuchereria bancrofti-*specific circulating antigen, and (ii) the prevalence of delayed type hypersensitivity (DTH) responses to *Leishmania* measured by leishmanin skin test (LST).

**Results:**

In this study, a total of 930 volunteers between the age of 18 and 65 were included from the two endemic areas of Kolokani and Kolondieba. In general, in both areas, filarial infection was more prevalent than *Leishmania* infection with an overall prevalence of 15.27% (142/930) including 8.7% (81/930) for *Mansonella perstans* and 8% (74/930) for *Wuchereria bancrofti-*specific circulating antigen. The prevalence of *Leishmania major* infection was 7.7% (72/930) and was significantly higher in Tieneguebougou and Bougoudiana (15.05%; 64/425) than in Boundioba (2.04%; 8/505) (*χ*^2^ = 58.66, *P* < 0.0001). Among the filarial infected population, nearly 10% (14/142) were also positive for *Leishmania* with an overall prevalence of co-infection of 1.50% (14/930) varying from 2.82% (12/425) in Tieneguebougou and Bougoudiana to 0.39% (2/505) in Boundioba (*P* = 0.0048).

**Conclusion:**

This study established the existence of co-endemicity of filarial and *Leishmania* infections in specific regions of Mali. Since both filarial and *Leishmania* infections are vector-borne with mosquitoes and sand flies as respective vectors, an integrated vector control approach should be considered in co-endemic areas. The effect of potential interaction between filarial and *Leishmania* parasites on the disease outcomes may be further studied.

## Background

Neglected tropical diseases (NTDs) are among chronic, disabling, and disfiguring diseases that occur most commonly in the setting of extreme poverty, especially among the rural poor and some disadvantaged urban populations having important socioeconomic impact [1, 2].

Filarial infections are chronic debilitating infections mostly caused by the mosquito-borne filarial nematodes, *Wuchereria bancrofti*, *Brugia malayi* and *Brugia timori* [1]. Worldwide, more than one billion people are at risk of infection for filariasis and 120 million people in the tropical and subtropical areas of the world are infected with parasites causing lymphatic filariasis (LF) [3]. The majority of those at risk of infection live in South-East Asia and Africa [4]. *Wuchereria bancrofti* (*W*. *bancrofti*) and *Mansonella perstans* (*M. perstans*) are the two major filarial infections found in Mali [5]. Before the mass drug administration (MDA), and based on the immunochromatographic card test (ICT), all districts were shown to be endemic for LF with an overall prevalence of 7.07% (ranging from 1% in the north to 18.6% in the south of Mali) [6]. Furthermore, the parasitological and entomological data from previous studies confirmed the districts of Sikasso in the South Sudan Savanna area and Kolokani in the North Sudan Savanna area as two areas of high *W. bancrofti* transmission [7].

Leishmaniasis is a vector-borne neglected disease transmitted to the host via the bite of *Leishmania*-infected female phlebotomine sand flies. Depending on the *Leishmania* species, humans can develop visceral or cutaneous forms of the disease. In the Old World (Africa, Europe and Asia), cutaneous leishmaniasis (CL) is characterized by skin lesions that can develop into unsightly scars, typically on the face and extremities. Lesions can heal spontaneously within several months, persist or relapse as chronic non-healing ulcers, or develop into complicated CL defined as mucosal lesions, nodular lymphangitis, and cutaneous dissemination (in immunocompromised individuals) [8]. Leishmaniasis is prevalent in more than 90 countries with an estimated 1.3 million new cases every year worldwide, and 20,000 to 30,000 deaths annually [9]. Furthermore, CL is the most reported form of this disease in some West African countries including Guinea Bissau [10, 11], Senegal [12, 13], Niger [14], Burkina Faso [15], Mali [16, 17] and Ghana [18, 19]. In Mali, *Leishmania major* (*L*. *major*) is the only species of *Leishmania* known to cause CL [20, 21] with *Phlebotomus duboscqi* (*P*. *duboscqi*) as the main sand fly vector in the country [21]. In 2009, a leishmanin skin test (LST) survey showed a high positivity rate of LST (45.4%) in Kemena and Sougoula, two villages in the central part of Mali [22]. The prevalence of *L. major* infection in *P. duboscqi* sand fly females was 2.7% in central Mali, confirmed by sequence alignment of a subset of polymerase chain reaction (PCR) products from infected sand flies; this vector is found in areas endemic for LF in Mali [21].

Since both filarial and *Leishmania* infections are vector-borne diseases with mosquitoes and sand flies as respective vectors, understanding the prevalence of their concurrent distribution may guide the disease control programme for evidence based integrated vector control approaches in co-endemic areas. It also contributes to an understanding of the significance of potential interaction between filarial and *Leishmania* parasites on the disease outcomes and may be further studied.

This study aimed to assess the prevalence of co-endemicity between the filarial parasites (*W. bancrofti* and *M. perstans*) and *L. major* parasite in Mali. To investigate the overlap between the two infections, we conducted a cross-sectional study in two ecologically distinct regions: in the Kolokani district (North Sudan Savanna area) and in the district of Kolondieba (South Sudan Savanna area).

## Methods

### Study sites

**Fig. 1:**
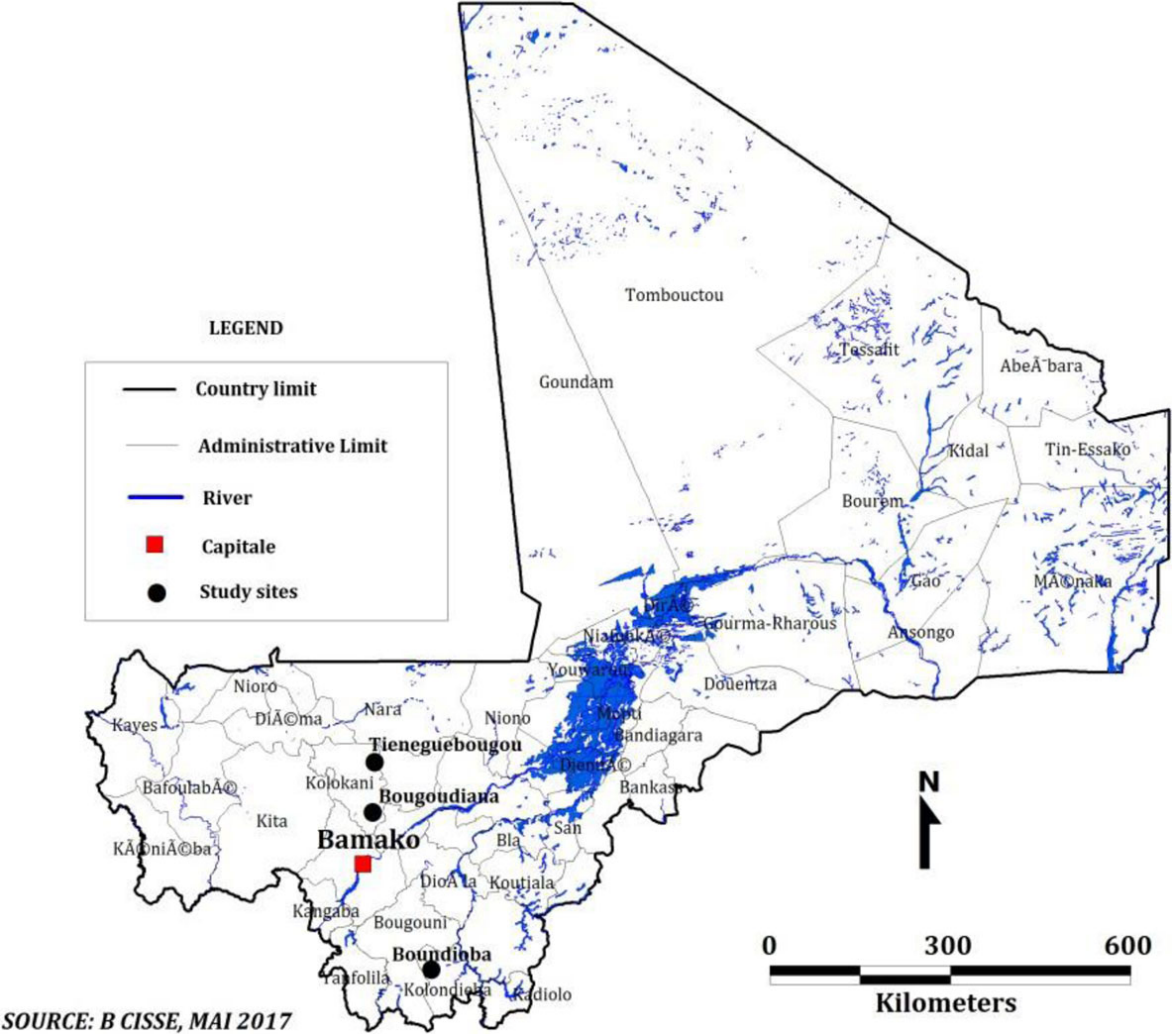
Study sites location. The dots represent the study villages

The study was conducted in the villages of Tieneguebougou and Bougoudiana in the Kolokani district (North Sudan Savanna area) and Boundioba in the district of Kolondieba (South Sudan Savanna area) (Fig. 1). The choice of these villages was based on historical data from previous studies of filariasis [7] where the prevalence of *W. bancrofti* infection was shown to be 48.3%, and co-infection with *M. perstans* to be 40.7% [23]. Notably, all regions in Mali are endemic for LF [6]. Cutaneous leishmaniasis was reported in the neighboring districts of Kolokani and Baroueli [22] but not in the south areas of Kolondieba.

A complete census, including the name, age, sex, and profession of all inhabitants, was performed in the study villages prior to the parasitological assessment. The population size of the villages varied from 1521 inhabitants in Tieneguebougou and Bougoudiana, to 3168 inhabitants in Boundioba. All volunteers aged 18–65 years who presented for evaluation were included in the study.

### Study design

**Fig. 2:**
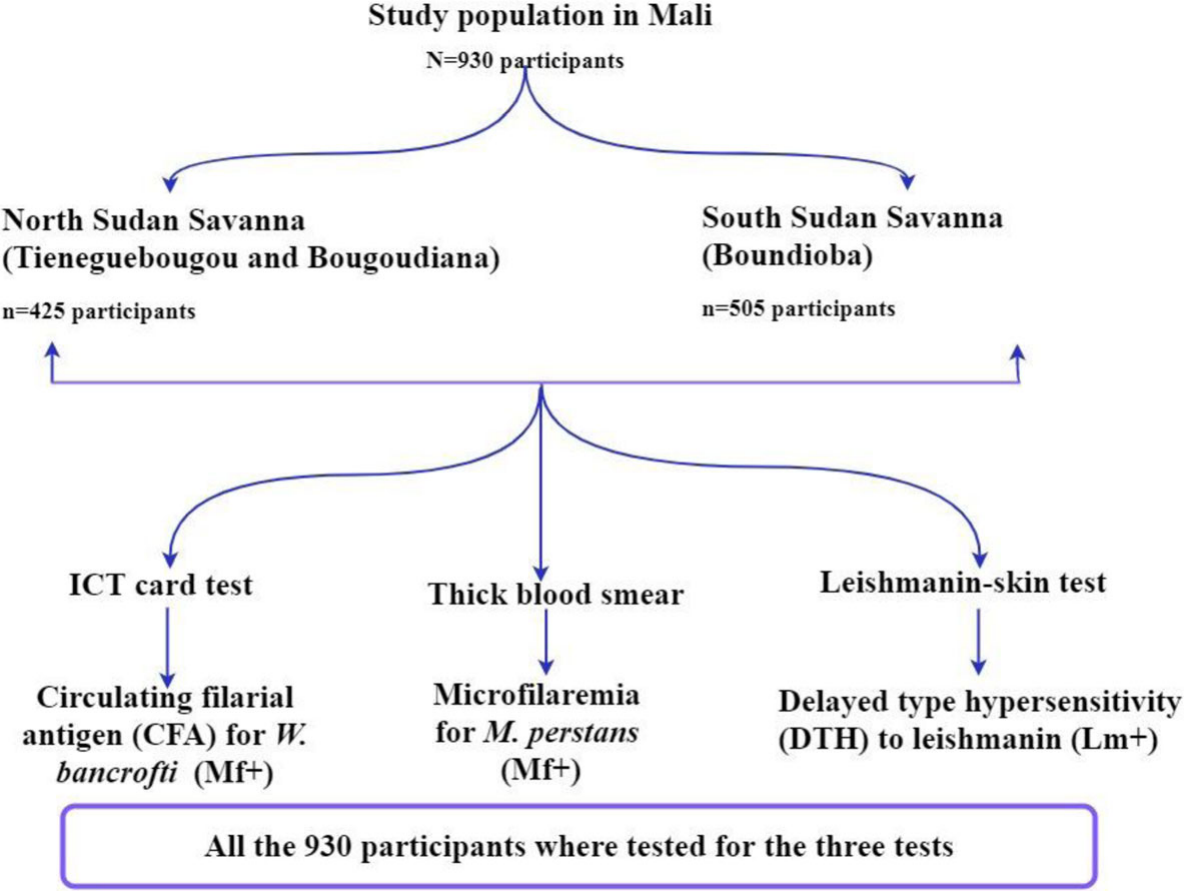
Study design. *Abbreviations*: Mf+, ICT positive for *Wuchereria bancrofti* or thick blood smear positive for *Mansonella perstans* (= filarial infection); Lm+, LST positive for *Leishmania major* (= *Leishmania* infection)

A cross-sectional survey was performed from June 2014 in the three study villages to May 2015 to assess (i) the prevalence of the two major filarial infections and (ii) the prevalence of coincident delayed type hypersensitivity (DTH) responses to leishmanin as measured by skin test. The Fig. 2 shows the study participants distributions between the 2 study areas (North and South Sudan Savanna) and the different tests performed.

### Clinical assessment at inclusion

All volunteers underwent a brief interview and an examination targeting the main signs and symptoms of the two study diseases. For LF, hydrocele and elephantiasis were researched through the interview (hydrocele) and the examination (elephantiasis). Leishmaniasis-related wounds were checked through both the interview and the examination.

### Laboratory evaluations/assays

#### Thick blood smear

Finger-prick and venous blood samples were collected using a calibrated thick blood smear (60 μl), obtained during the daytime. The blood was drawn onto a microscope slide, dried and stained with 10% Giemsa using standard procedures. The stained smears were examined using a light microscope with a 10× objective for the detection of *M. perstans* microfilariae (see Fig. 2).

#### Immunochromatographic card test (ICT)

The ICT Filariasis test used is a rapid-format filarial antigen test that was developed by ICT diagnostics (BinaxNow® Filariasis test, Alere Inc., Scarborough, USA). The test is designed for detection of soluble *W. bancrofti* antigens that circulate in the blood of infected humans. The test was performed according to the manufacturer’s instructions.

#### Leishmanin skin test (LST)

LST is an indication of previous exposure to *Leishmania* parasites. LST material is supplied as sterile aqueous 2.5 ml suspensions, with each ml containing 6 × 106 killed *L. major* promastigotes (strain MRHO/IR/75/ER) in phosphate buffered saline and 0.01% thimerosal, pH 7.0–7.1 (stable for 5 years when stored at 2–8 °C). LST has a very good sensitivity and specificity varying from 80% to 100% [24]. The leishmanin (0.1 ml) was injected intra-dermally in the left forearm. Readings were taken 48 to 72 h after the injection using a ball point pen to determine the size of the induration. Measurements with a diameter greater than 5 mm were considered positive [25].

### Data management and analysis

Data were recorded on excel spreadsheet and statistical analysis was performed using GraphPad Prism version 7.03 (GraphPad Software Inc., San Diego, CA, USA). Statistically significant differences between proportions were analyzed using Pearson’s chi-square test.

## Results

### Characteristics of the study population in the two ecologically distinct settings

A total of 930 study subjects (230 in Tieneguebougou, 195 in Bougoudiana, and 505 in Boundioba) of both genders aged 18 to 65 years old were included in the study. In each village, the number of female participants was higher with a female to male sex ratio of 1.6 in Tieneguebougou and Bougoudiana, and 3.5 in Boundioba. The median age in Tieneguebougou and Bougoudiana, and Boundioba was 35 (range 25–48) and 32 (range 22–44.5) years respectively.

### Prevalence of filarial and *Leishmania* parasite infections

**Fig. 3:**
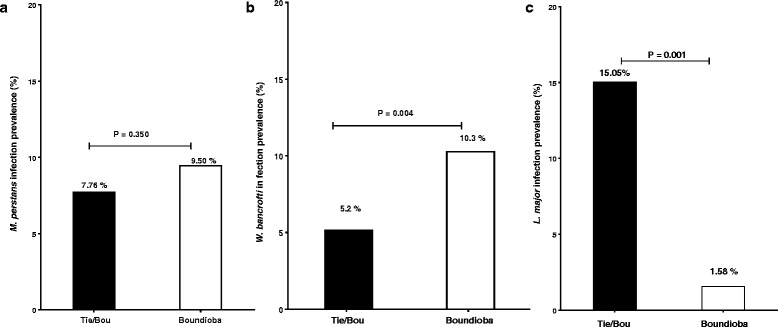
Prevalence of *M. perstans * (**a**) *W. bancrofti* (**b**) and *L. major* (**c**) infections among study populations in Tieneguebougou and Bougoudiana, and Boundioba. *Abbreviation*: Tie/Bou, Tieneguebougou and Bougoudiana

For filarial infections, we found a prevalence of 8.70% (81/930) for *M. perstans* infection and 8.00% (74/930) for *W. bancrofti* infection*.* Furthermore, the prevalence for *M. perstans* and for *W. bancrofti* increased gradually with age (Table 1). The prevalence of *M. perstans* microfilaremia in Tieneguebougou and Bougoudiana, and Boundioba was 7.76% (33/425) and 9.50% (48/505), respectively (Fig. 3). These prevalences were comparable between the two sites (*χ*^2^ = 0.88, *df* = 1, *P* = 0.35). However, the positivity rate of *W. bancrofti* circulating filarial antigen (CFA) detected by ICT was significantly higher in Boundioba, 10.30% (52/505) as compared to Tieneguebougou and Bougoudiana, 5.20% (22/425) (*χ*^2^ = 8.26, *df* = 1, *P* = 0.004) (Fig. 3).

**Table 1:** Prevalence of *M. perstans*, *W. bancrofti* and *L. major* infections among the participants according to sex and age group

	Total examined	*M. perstans* ^a^	*W. bancrofti* ^b^	*L. major* ^c^
	(*N*)	Positive *n* (%)	Positive *n* (%)	Positive *n* (%)
Sex				
Male	274	27 (9.9)	20 (7.3)	28 (10.2)
Female	656	54 (8.2)	54 (8.2)	44 (6.7)
Age group				
18–27	325	24 (7.4)	17 (5.2)	12 (3.7)
28–37	238	16 (6.7)	21 (8.8)	18 (7.6)
38–47	165	15 (9.1)	17 (10.3)	15 (9.1)
48–57	125	17 (13.6)	12 (9.6)	17 (13.6)
≥ 58	77	9 (11.7)	7 (9.1)	10 (13.0)
Total	930	81 (8.7)	74 (8.0)	72 (7.7)

^a^*Mansonella perstans* (from microfilaremia prevalence)

^b^*Wuchereria bancrofti* (from circulating filarial antigen prevalence)

^c^Leishmaniasis diagnosis was done using Delayed Type Hypersensitivity (DTH) to Leishmanin Skin Test (LST)

The overall prevalence for *Leishmania* infection (indicated by positive LST) was 7.74% (72/930) with 10.21% (28/274) in men and 6.70% (44/656) in women (*χ*^2^ = 3.33, *df* = 1, *P* = 0.067) (Table 1). This prevalence gradually increased with age and was significantly higher in Tieneguebougou and Bougoudiana (15.05%; 64/425) as compared to Boundioba, (1.58%; 8/505) (*χ*^2^ = 8.26, *df* = 1, *P* < 0.0001) (Fig. 3). None of the study volunteers showed clinical signs or symptoms related to the study diseases.

### Prevalence of filarial and *Leishmania* co-infections

The overall prevalence of concurrent infections (Mf^+^/LST^+^) among individuals was 1.51% (14/930), with 6.24% (58/930) Mf^−^/LST^+^ and 13.76% (128/930) Mf^+^/LST^−^. Of the 14 cases of co-infection, 12 of 425 subjects (2.82%) were from Tieneguebougou and Bougoudiana, and 2 of 505 subjects (0.40%) were from Boundioba. There were more co-infections in men than women (ratio of 8/6).

## Discussion

In this study, we show that *W. bancrofti* infection prevalences were 7.69% in Tieneguebougou, 3.04% in Bougoudina, and 10.30% in Boundioba (Fig. 3) suggesting that bancroftian filariasis is still a public health problem in these regions of Mali. The prevalences of microfilaremia for *M. perstans* were 11.79%, 4.35% and 9.50%, for Tieneguebougou, Bougoudiana and Boundioba, respectively, suggesting that this parasite is currently the most prevalent filarial infection in Mali. This study further confirms that *M. perstans* is widespread througout the country and supports previous studies done in this region [26]. Further studies need to be performed to evaluate the clinical significance of mansonellosis in humans. Together, these results suggest that filarial infections as well as *Leishmania* infections are still endemic in Kolokani (Tieneguebougou and Bougoudiana) and Kolondieba (Boundioba) districts of Mali.

Within the 142 infected volunteers with at least one of the two filarial infections (*W. bancrofti* and *M. perstans*), a co-infection prevalence of 9.15% (13/142) was observed. This observation indicates the endemicity of both filarial infections in the study areas. This co-infection was also observed in a neighboring area of Kolokani before any mass drug administration with a high prevalence of 48.3% (69/143) [23]. A high prevalence of *M. perstans* infection was also reported in other countries such as Senegal [27], Nigeria [28], Ghana [29], Burkina Faso [30], Gabon [31], as well as Cameroon in Central Africa [32]. Moreover, previous studies reported symptoms such as symptomatic hypereosinophilia related to *M. perstans* microfilaremia [33].

While several mass drug administration campaigns have been implemented since 2005 in Mali, the current study suggests that filarial infections persist regardless of previous implementations. Furthermore, despite a drastic decrease [34] due to nine annual MDA rounds in the study area, LF prevalence is still higher than the 1% endemicity threshold. These findings are important not only to the health authorities involved in filariasis elimination programme’s efficacy assessments, but also to the scientific community for providing useful information to achieve elimination by 2020 [35].

Another interesting finding in our study was the higher positivity rate of LST in Kolokani (Tieneguebougou and Bougoudiana) compared to Kolondieba (Boundioba) (*P* < 0.0001) (Fig. 3). Our previous data indicate that sand fly anti-saliva antibody levels are similar across these two areas [36]. The differences may be due to a higher infection rate of sand flies in Kolokani area that is contiguous to two villages in Central Mali. In these areas, CL has an estimated infection rate of 2.7% in the female population of *P. duboscqi* [21]. The difference in infection rates between the two areas could be further explained by the distribution of reservoir mammals, climate, immunity, or even genetic background [37].

**Fig. 4:**
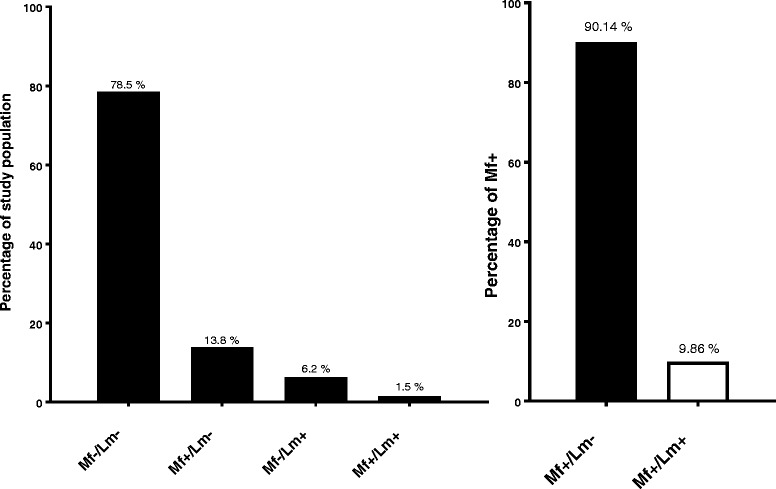
The prevalence of filarial and *Leishmania* co-infection in Tieneguebougou and Bougoudiana, and Boundioba. *Abbreviations*: Mf+, positive filarial infection; Mf-, negative filarial infection; Lm+, positive *Leishmania* infection; Lm-, negative *Leishmania* infection

Epidemiological studies suggest that CL is widely distributed throughout Mali [38]. In fact, the prevalence of *L. major* infection is as high as 45% within individuals residing in central Mali (Kemena, 45% LST-positive and Sougoula 20% LST-positive) [22]. Furthermore, our study indicates that 1.5% of the total population in the two study districts (Kolokani and Kolondieba) are co-infected with *Leishmania* and filariae (Mf^+^/Lm^+^) (Fig. 4) suggesting the occurrence of poly-parasitism in these regions. To our knowledge this is the first study reporting a prevalence rate of filarial/*Leishmania* (*L. major* and *W. bancrofti* and/or *M. perstans*) co-infection in Mali. A co-infection of filariasis and visceral leishmaniasis (*W. bancrofti* and *L. donovani*) has been also reported in a resident of Bihar, India [39]. Other studies have established co-infections with poly-parasitism (malaria and filariae) in Mali [5, 40], as well as other parts of Africa (loiasis and onchocerciasis in Nigeria, and schistosomiasis and hookworm infection in Kenya) [41, 42].

Finally, co-infection may severely impact the immune response. How chronic filarial infections alter the host’s immune response to *Leishmania* [43], to further facilitate *L. major* infection in Mali, is currently under investigation.

## Conclusions

Filarial and *Leishmania* infections are still endemic in Kolokani (Tieneguebougou and Bougoudiana) and Kolondieba (Boundioba) districts of Mali suggesting the co-endemicity of these two diseases. Since both infections are vector borne diseases with mosquitoes and sand flies as respective vectors, an integrated vector control approach should be considered in co-endemic areas. While currently there are no leishmaniasis control programmes in Mali, filarial and *Leishmania* co-infection might be of great importance to the neglected tropical diseases control programme. More studies need to be undertaken to assess the effect of co-infection on the host immune response that may influence the outcome of disease.

## Abbreviations

CFA: Circulating filarial antigen
CL: Cutaneous leishmaniasis
DTH: Delayed type hypersensitivity
ICT: Immunochromatographic test
LF: Lymphatic filariasis
LST: Leishmanin skin test
MDA: Mass drug administration
NTDs: Neglected tropical diseases
